# ZnO/AC recovered from PET bottles *via* pyrolysis for the hybrid treatment (adsorption/photocatalysis) of BB41 dye in aqueous media

**DOI:** 10.1039/d6ra00311g

**Published:** 2026-05-05

**Authors:** Nadjib Dahdouh, Haroun Hafsa, Badreddine Djemmali, Amina Ouarghi, Imane Kezrane, Toufik Chaabane, Venkataraman Sivasankar, Mohammed Kebir

**Affiliations:** a Reaction Engineering Laboratory, University of Science and Technology HouariBoumediene B.P.32, El-Alia Bab-Ezzouar Algeria nadjibdahdouh@outlook.com; b Scientific and Technical Center of Research in Physical and Chemical Analysis CRAPC BP384 Bou-Ismail Tipaza 42004 Algeria; c Department of Civil Engineering, School of Engineering, Nagasaki University 1-14 Bunkyo-machi Nagasaki 852 8521 Japan

## Abstract

Plastic waste continues to increase on land and poses a critical danger to human life, which is why our contribution addresses this issue. Waste PET bottles were collected and subjected to pyrolysis to produce activated carbon, which was then doped with ZnO to form a ZnO/AC(PET) heterostructured composite. The deposition of ZnO nanoparticles on activated carbon was confirmed using different characterization techniques, such as SEM, XRD, and FTIR spectroscopy, which demonstrated the effectiveness of our doping method. High-performance ZnO/AC(PET) was used for the hybrid treatment of solutions synthetically colored with the textile dye blue basic 41. Adsorption and solar photocatalysis are the two techniques chosen in our study. The mass effect of the hybrid material and the effect of the initial concentration of the BB41 dye were evaluated. A maximum adsorption capacity of 561.80 mg g^−1^ was recorded for ZnO/AC(PET) due to the pores present on the surface of the activated carbon. After the adsorption step, the treated solutions were further exposed to solar irradiation for a secondary treatment, achieving a removal efficiency of up to 94.44%. The process was optimized by identifying the model that best fits the experimental data and determining the main kinetic mechanism of the photocatalytic degradation of the BB41 dye using first and second-order models. The findings demonstrated that, given the high correlation coefficients, the first-order model offered the best match. This work highlights the valorization of PET waste into ZnO/activated carbon, which shows good affinity for BB41 dye and functions as both an adsorbent and a photocatalyst.

## Abstract 2

Plastic waste-derived activated carbon and ZnO-doped composite were developed for efficient wastewater treatment through synergistic adsorption–photocatalysis for enhanced pollutant removal.

## Graphical abstract

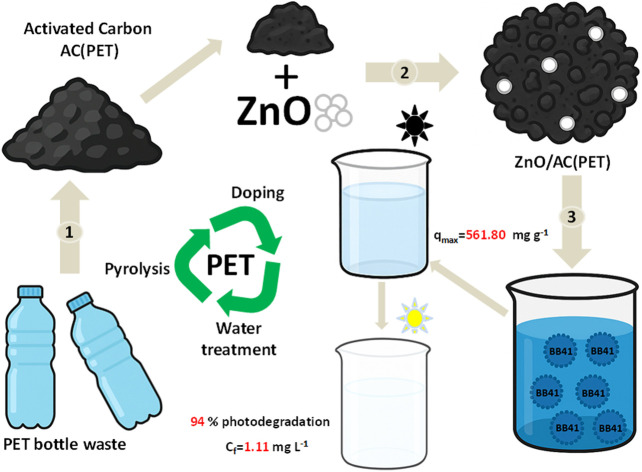

## 1. Introduction

Driven by the pursuit of greater comfort, human activities generate various non-biodegradable materials that persist in the environment for centuries and pose serious risks to future generations due to their complex composition. This contradicts the sustainable development strategy and plastic is one such material. It has become an essential aspect of human life. This material is indispensable in many objects. Almost all objects we use in daily life contain plastic. The plastic industry continues to produce large quantities of plastics to meet growing demand, while researchers are developing new materials because of their low cost and wide range of applications. Currently, 300 types of plastics are produced, of which around 60 are the most demanded ones.^1^ Plastics are not innocuous; they contain a number of harmful substances that can spread as microplastic pollutants, which have been associated with adverse health effects.^2,3^ Plastic polymers release dioxins, which are deadly organic pollutants that harm the nervous system, cause cancer, and hinder the development of the reproductive system.^4^ Microplastics have several negative effects on human, birds, the marine ecosystems, agriculture and soil.^5^ Studies provide significant data on plastic production; however, a major concern is that a large proportion of these materials ultimately becomes waste.^6–8^ This situation represents a serious environmental concern. Poly (ethylene terephthalate) (PET) is one of the most widely produced polymers globally, with its use dominating the single-use packaging sector, including bottles and textiles. In 2020, PET accounted for a significant portion of single-use bottles, yet the global recycling rate remained low at around 26.6%, highlighting the need for improved recycling practices and sustainable management of PET waste.^9^ Environmentalists are worried about the future of PET masses manufactured by the industry and have announced an alert to save fauna and flora. Studies show that more than 400 billion bottles of PET are produced worldwide each year, and almost half (46%) are used as water packaging.^10^ By 2021, this production could increase by 20%. Scientists continue to publish papers on the dangers of plastic waste and seek solutions that are both environmentally friendly and cost-effective.^11,12^ The pyrolysis process is one of the recycling techniques that can reduce and valorize significant quantities of PET waste present in the environment. The pyrolysis of plastic waste involves burning it at high temperatures under a controlled atmosphere (inert gas)^13,14^ to produce active coal that is widely used in wastewater treatment due to its affinity for pollutants in water.^15–18^ The process of employing pyrolysis-derived activated carbon is called adsorption. By interacting the activated carbon with aqueous hazardous chemicals, the activated carbon's pores allow the molecules to be adsorbed.^19^ This method is commonly used in various fields to manage effluents before they are released into the receiving environment. These include natural and wastewater management,^20^ chemicals,^21^ petrochemicals,^22^ pharmaceuticals,^23^ food processing,^24^ textiles,^25^ paper manufacturing,^26^ mining and metallurgy.^27^ However, there are other methods of managing toxic effluents, such as photocatalysis.^28^ This latest method that uses the immense solar energy is also a conventional method and one of the advanced oxidation processes developed in recent years for the degradation of pollutants in aqueous medium.^29^ Among the materials used in photocatalysis, zinc oxide (ZnO) presents several advantages.^30–33^ Recent developments have demonstrated that nano-ZnO can be employed as a heterogeneous catalyst due to its low cost and environmental benefits, which include minimal reaction time, low corrosion, waste reduction, catalyst recyclability, and ease of transportation and disposal.^34^ Because of its special qualities, including a large free-exciton binding energy that allows excitonic emission processes to persist at or even above room temperature, a direct and wide band gap in the near-UV spectral region, strong oxidation ability, and good photocatalytic properties, ZnO has become a leading candidate in green environmental management systems.^35^ The doping of activated charcoal by a photocatalyst is also effective and improves the ability to degrade pollutants in wastewater, especially dyes.^36–38^ The latter are widely used in the textile industry, and as has been shown by researchers, textile dyes are harmful and can present great damage to fauna and flora.^39–41^ The aquatic reservoir's pH, biochemical oxygen demand (BOD), total dissolved solids (TDS), total suspended solids (TSS), and chemical oxygen demand (COD) are subject to change when the effluent is discharged.^42^ This study aims to examine the capacity of activated carbon doped with ZnO for the discoloration of water synthetically colored by BB41. The activated carbon used in this study was derived from the pyrolysis of PET plastic bottles. The activated carbon was then doped with zinc oxide. The resulting heterosystem was used to remove a BB41 cationic textile dye. The solutions contaminated with BB41 dye underwent adsorption in the dark, followed by photocatalysis upon exposure to sunlight. Finally, encouraging results were obtained and are presented in our study.

## 2. Experimental

### 2.1. Reagents and chemicals

High-purity reagents were used without any prior purification. Zinc acetate dihydrate, Zn (CH_3_CO_2_)_2_·2H_2_O (99%), was supplied by Sigma-Aldrich. Sodium hydroxide NaOH (85%) was purchased from PA Panreac. *N*,*N*-dimethylformamide (DMF) C_3_H_7_NO (99.8%) was supplied by Sigma-Aldrich. Sulfuric acid (H_2_SO_4_) (96%) was provided by Carlo Erba. Finally, Basic Blue 41 C_20_H_26_N_4_O_6_S_2_ (98%) was obtained from TDA SPA, an Algerian company specializing in the textile industry.

### 2.2. Activated carbon synthesis procedure

PET bottles were collected from a street in Algiers. They were thoroughly washed with soap and tap water to remove dust and impurities and then cut into 1 cm × 1 cm pieces. The samples were rinsed several times with distilled water for later use. PET pellet waste (20 g) was impregnated with a sulphuric acid solution (impregnation ratio = 1) and stirred at 85–95 °C for 6 h to allow diffusion of the reagents. After evaporation at 110 °C for 15 h, the treated PET was pyrolysed in a tube furnace under an inert atmosphere (N_2_, 50 mL min^−1^). Heating was carried out in two stages: 400 °C (2.5 °C min^−1^) for 1 h and then 800 °C for a further 1 h before cooling. The activated carbon obtained was washed with water to remove impurities and sulphur compounds, followed by Soxhlet extraction with hot distilled water to a neutral pH. Finally, it was dried overnight at 110 °C. The experimental procedure was carried out following the protocol described by Mohammed Adibfar and others.^43^

### 2.3. Synthesis of the ZnO/activated carbon (PET) photocatalyst composite

In this study, a composite based on activated carbon and zinc oxide (ZnO) was synthesized using an *in situ* intercalation method. The aim was to prepare a ZnO/AC hybrid material that can be used for adsorption and photocatalysis applications. The procedure followed was established by Boutra and Trari.^44^ Activated carbon, sieved to a size of 200 micrometers, was used as a support for the incorporation of nanometric ZnO. To do this, 2 g of activated carbon was dispersed in a solution containing 400 mg of zinc acetate dihydrate dissolved in 50 mL of dimethylformamide (DMF). The suspension was sonicated for 3 hours to ensure homogeneous dispersion. Next, 100 mL of an aqueous NaOH solution (0.1 M) was added with continuous stirring for 1 hour. The resulting composite was purified by centrifugation, followed by several stages of dispersion in alcohol. After drying under vacuum at 75 °C for 4 hours, it was calcined at 200 °C for 2–3 hours. The final material, an activated ZnO/carbon composite, was then used for adsorption and photocatalysis tests.

### 2.4. Preliminary tests on cationic and anionic dyes

Preliminary experiments were conducted using both cationic and anionic dyes to evaluate the potential applicability of the ZnO/AC(PET) composite. Basic Blue 41 (cationic) and methyl orange (anionic) were tested under identical conditions. Although BB41 showed significant adsorption and photocatalytic degradation, methyl orange exhibited no measurable adsorption or photocatalytic activity, highlighting that the composite was more effective for the cationic dyes.

### 2.5. Preparation of BB41 solutions

A stock solution of the basic dye BB41, with a concentration of 500 mg L^−1^, was prepared by dissolving 0.5 g of BB41 powder in 1 L of distilled water. From this, solutions of the desired concentrations were prepared in order to study the elimination of BB41 by a hybrid system combining adsorption and photocatalysis.

### 2.6. Adsorption/photocatalysis experiments

All experiments were carried out in a batch reactor at the initial solution pH without subsequent adjustments. One of the objectives of this work is to evaluate the adsorption and photocatalytic performance at the natural pH of the solution in order to minimize the use of additional chemicals such as NaOH and HCl. All experiments were performed at room temperature (25 ± 1.5 °C). This approach was adopted to better simulate the real conditions encountered in industrial wastewater treatment processes without the need for external thermal control while ensuring relatively stable experimental conditions.

#### 2.6.1. BB41 adsorption kinetics

To compare the efficiencies of AC and ZnO/AC(PET) by determining the equilibrium time and adsorption rate, an experiment was carried out by bringing 20 mg of the material into contact with 100 mL of a BB41 solution at an initial concentration of 20 mg L^−1^. The experiment was carried out at a neutral initial pH of approximately 7 and an ambient temperature of 25 °C while carefully keeping the system protected from light in order to avoid any photodegradation of the dye. In order to monitor the adsorption kinetics, samples were taken at regular time intervals, covering a range from 2 minutes to 180 minutes. Changes in the residual dye concentration were analyzed to determine the time required to reach adsorption equilibrium.

#### 2.6.2. Photocatalysis batch experiments

According to several studies carried out, photocatalysis takes place in two stages: adsorption and photodegradation under the effect of light.^45^ First, we began with the adsorption of BB41 on the ZnO/AC(PET) surface in the dark. The photodegradation of BB41 was studied after reaching the equilibrium time of adsorption, after which the solutions were exposed to sunlight for up to 180 min. The dose effect of modified ZnO/AC(PET) and the effect of the initial dye solution were studied. To determine the optimum dose of ZnO/AC(PET) for the photodegradation of BB41 dye, its dosage was varied from 5 to 25 mg for 180 min, using a solution concentration of 20 mg L^−1^. After optimizing the optimum dose of doped ZnO/AC(PET), the effect of the initial BB41 concentration was investigated at 20, 30, 40, 50, and 60 mg L^−1^.

#### 2.6.3. Determination of residual concentration

The residual concentrations of BB41 were determined using a previously established calibration curve. All absorbance measurements were performed in triplicate to ensure reproducibility. The absorbance of the solutions was measured using a SHIMADZU UV-1800 spectrophotometer, which provided reliable readings with high accuracy under these conditions. This spectrophotometric approach allowed for the precise quantification of the dye concentration after adsorption and photodegradation. Scans were carried out to analyse the residual solutions of BB41, and the absorbance of the solutions was measured after adsorption and after photocatalysis at a wavelength of 610 nm. The precision of the quantification is ensured by the linearity of the calibration curve and the reproducibility of the measurements. The adsorption and photodegradation efficiencies were determined using the following equation:^46^1
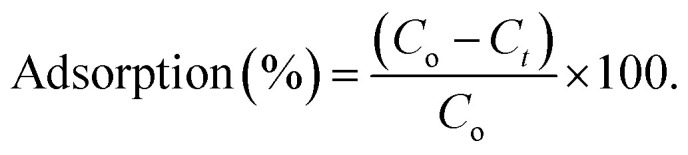


The maximum adsorption capacity was determined using the following formula:^46^2
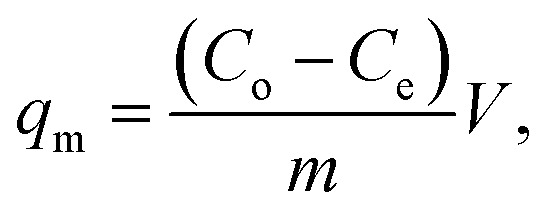
where *C*_o_, *C*_*t*,_ and *C*_e_ are the initial, residual at time, and equilibrium BB41 dye concentrations, respectively. *V* is the volume of the solution, and *m* is the mass of the composite ZnO/AC (PET).

### 2.7. Structural and surface characterization of ZnO/AC(PET) composite

To gain a deeper understanding of the reaction mechanism involved in the photodegradation of BB41 in the presence of the ZnO/AC(PET) hybrid material, an in-depth characterization of the materials was carried out. AC, ZnO, and the ZnO/AC composite were analyzed using several physico-chemical characterization techniques. A PAnalytical diffractometer was applied for X-ray diffraction (XRD) analysis, allowing for the identification of the crystalline phases present and the determination of the crystallographic structure of the materials. The FTIR spectra were recorded using a Bruker Vertex 70 spectrometer in the wavelength range of 4000–400 cm^−1^, with a scan speed of 4 cm^−1^ per second. To better understand the mechanisms of adsorption and photodegradation, the morphology and surface structure of the materials were examined using scanning electron microscopy (SEM) with Quanta 650.

### 2.8. Determination of pH_PZC_

The point of zero charge (pH_PZC_) of the solid material ZnO/AC was evaluated using the pH drift approach, which determines the pH at which the catalyst surface exhibits a neutral net charge. A 50 mL solution of 0.01 N NaCl was prepared and divided into multiple beakers. The initial pH (pH_0_) of each solution was adjusted between 2 and 12 using 0.01 N HCl or 0.01 N NaOH. Subsequently, 0.1 g of solid ZnO/AC was added to each beaker, and the mixtures were continuously stirred for 24 hours to establish adsorption–desorption equilibrium. After equilibration, the final pH (pH_f_) was measured using a calibrated pH meter. The pH_PZC_ was determined as the pH at which pH_0_ matched pH_f_, reflecting no net proton exchange due to surface charge neutralization.^47^

### 2.9. Active species in photocatalytic degradation

Identifying the primary reactive oxygen species (ROS) responsible for the photodegradation of BB41 dye is crucial to understanding reaction pathways. The main ROS involved typically include photogenerated electrons (e^−^), holes (h^+^), superoxide radicals (O_2_˙^−^) and hydroxyl radicals (OH˙). To elucidate their respective roles, a representative ZnO/activated carbon (PET) composite catalyst was employed. Under optimized experimental conditions, two sets of radical scavenging tests were performed using specific chemical quenchers to selectively inhibit each reactive species. Ethylenediaminetetraacetic acid disodium salt (EDTA) was utilized as a hole (h^+^) scavenger, isopropanol (ISP) was applied to quench hydroxyl radicals (OH˙), ascorbic acid (ASA) was applied to trap superoxide radicals (O_2_˙^−^) and potassium dichromate (K_2_Cr_2_O_7_) functioned as an electron (e^−^) scavenger.^48–50^ These scavenging studies provided insights into the dominant reactive species facilitating BB41 degradation and helped clarify the photocatalytic mechanism.

## 3. Results and discussion

### 3.1. Characterization of the ZnO/AC(PET) hybrid material

**Fig. 1:**
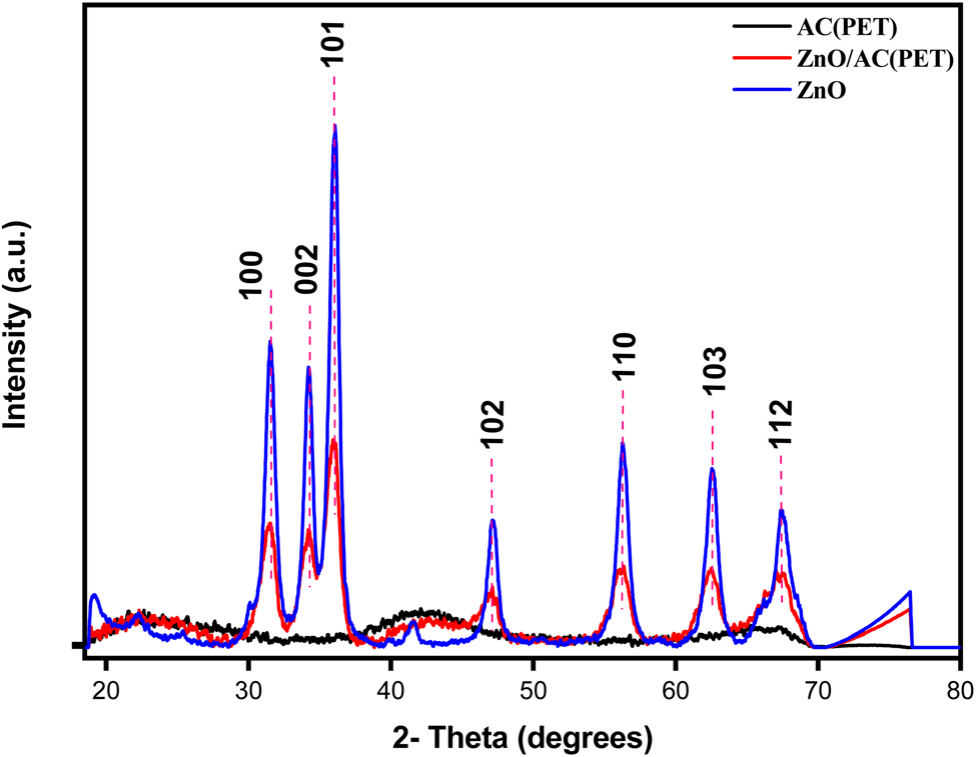
XRD patterns of AC(PET), ZnO and ZnO/AC(PET).

**Fig. 2:**
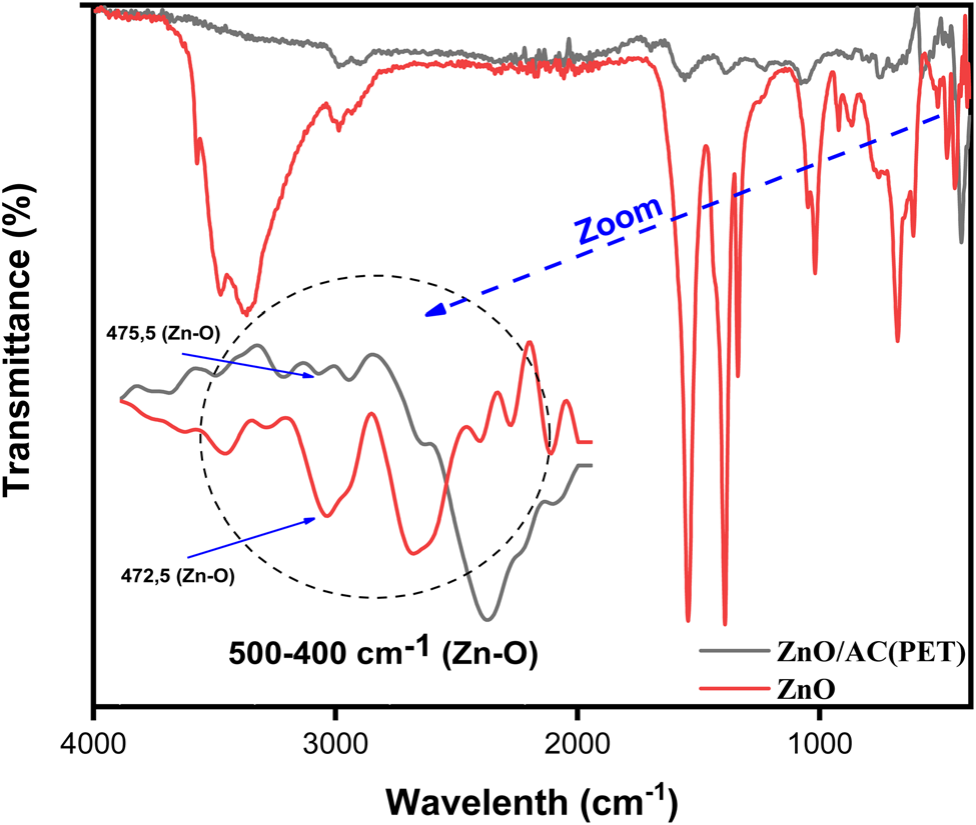
FTIR spectra of ZnO and ZnO/AC(PET) heterosystem.

**Fig. 3:**
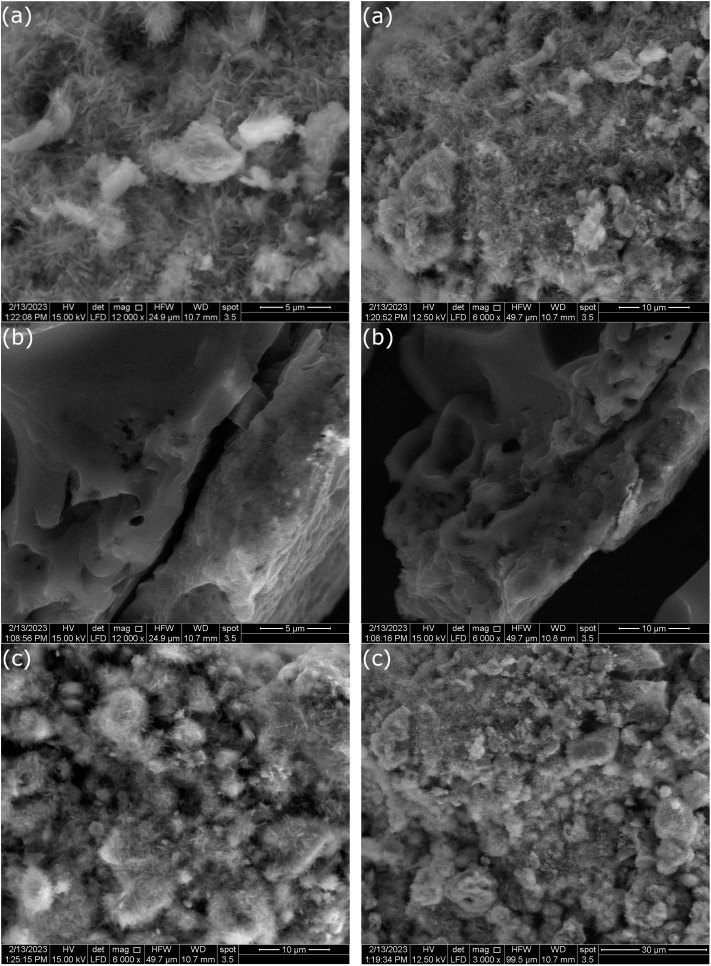
SEM images of ZnO (a), AC(PET) (b), and ZnO/AC(PET) composite (c).

The XRD spectra of ZnO, AC(PET) and ZnO/AC(PET) obtained are presented in Fig. 1. The seven characteristic peaks of ZnO, confirming its hexagonal wurtzite-type crystalline structure, appear at the following 2*θ* angles: 31.53°(100), 34.22°(002), 36.08°(101), 47.2°(102), 56.25°(110), 62.54°(103) and 67.4°(112). With a little shift at 2*θ* that could be caused by variations in structure or synthesis circumstances, the spectrum obtained displays the same distinctive peaks as those reported in the literature for ZnO wurtzite.^51^ The spectrum in Fig. 1 demonstrates the amorphous form of the activated carbon we produced by pyrolyzing PET waste. The X-ray diffractogram of activated carbon (PET) impregnated with ZnO shows the same characteristic peaks as crystalline ZnO but with lower intensity. This decrease in intensity may be attributed to the dispersion of ZnO particles on the amorphous surface of the activated carbon as well as the relatively low concentration of ZnO in the composite material. The FTIR spectrum of ZnO and ZnO/AC(PET) was recorded in the range 375–4000 cm^−1^, and it is illustrated in Fig. 2. In the FTIR spectrum of ZnO, a significant vibration band ranging from 400 cm^−1^ to 500 cm^−1^ is assigned to the characteristic stretching mode of the Zn–O bond. This has already been confirmed by G. Nagaraju *et al.*^52^ In the ZnO/AC(PET) spectra, the band is still discernible while being less strong and displaced, indicating that ZnO is present and interacts well with the carbon support. The peak at 472.5 cm^−1^ is due to the characteristic vibration of the Zn–O bond. This result is in agreement with the findings of V.P. Dinesh *et al.*^53^ A less intense and slightly shifted peak (475.5 cm^−1^) was observed in the ZnO/AC(PET) spectrum, suggesting the deposition of ZnO on the surface of the activated carbon. Fig. 3 displays the surface morphology of the scanning electron microscopy (SEM) images. ZnO nanoparticles (Fig. 3a) are agglomerated and represented in a hexagonal-pyramidal morphology. Fig. 3b shows an irregular structure with different pore sizes (from 7.14 µm to 58.07 µm) and many cavities due to the use of activated carbon. These cavities are due to the evaporation of the activating agent (H_2_SO_4_), which creates vacant spaces during activation. Such a structure was observed by A. Machrouhi *et al.*^54^ The morphology of activated carbon presents irregular cavities slightly modified after loading with an activation agent. Fig. 3c shows a homogeneous microstructure with small ZnO agglomerates. The SEM image clearly shows the distribution of ZnO nanoparticles on the surface of the activated carbon. This type of surface morphology is ideal for use in adsorption and photocatalysis. We also notice areas that are not covered. This suggests that the ZnO particles are deposited inside the pores of the activated carbon.

**Fig. 4:**
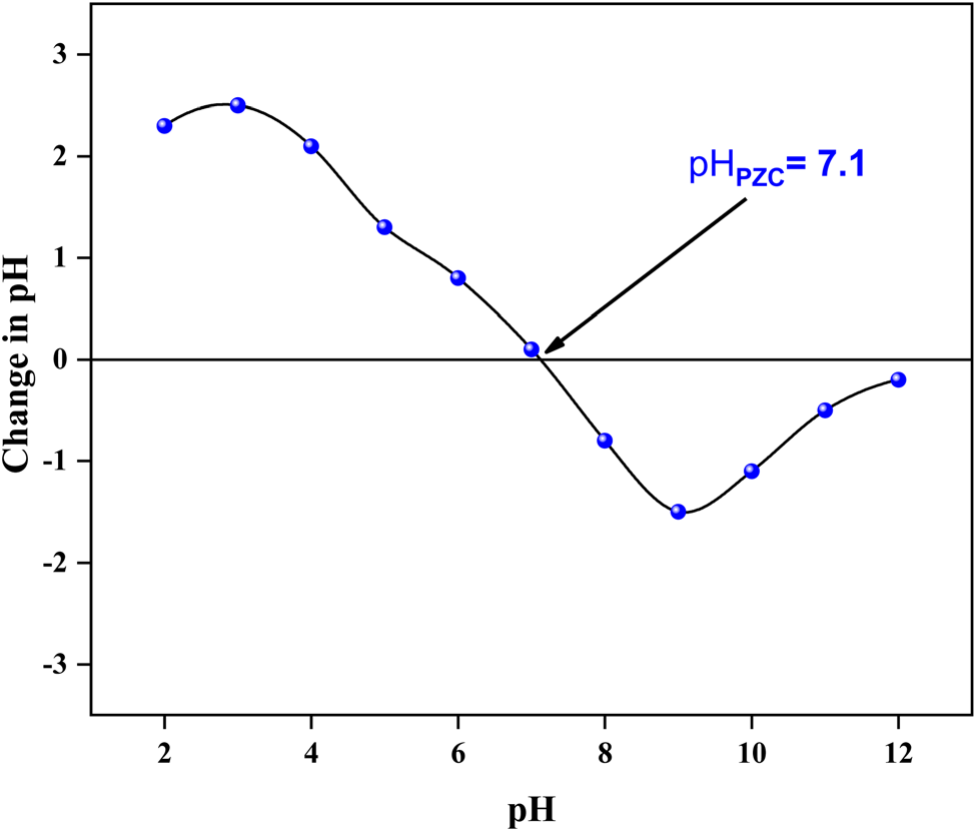
pH_PZC_ plot of the ZnO/AC composite.

For the ZnO/AC(PET) solid material, the surface charge profile as a function of pH indicates a pH_PZC_ of approximately 7.1. Below this pH value, the surface carries a net positive charge, whereas above it (pH > 7.1), the surface becomes negatively charged. This shift in surface charge significantly influences interfacial interactions and adsorption behavior during photocatalytic reactions (Fig. 4).

### 3.2. BB41 adsorption efficiency *versus* time

**Fig. 5:**
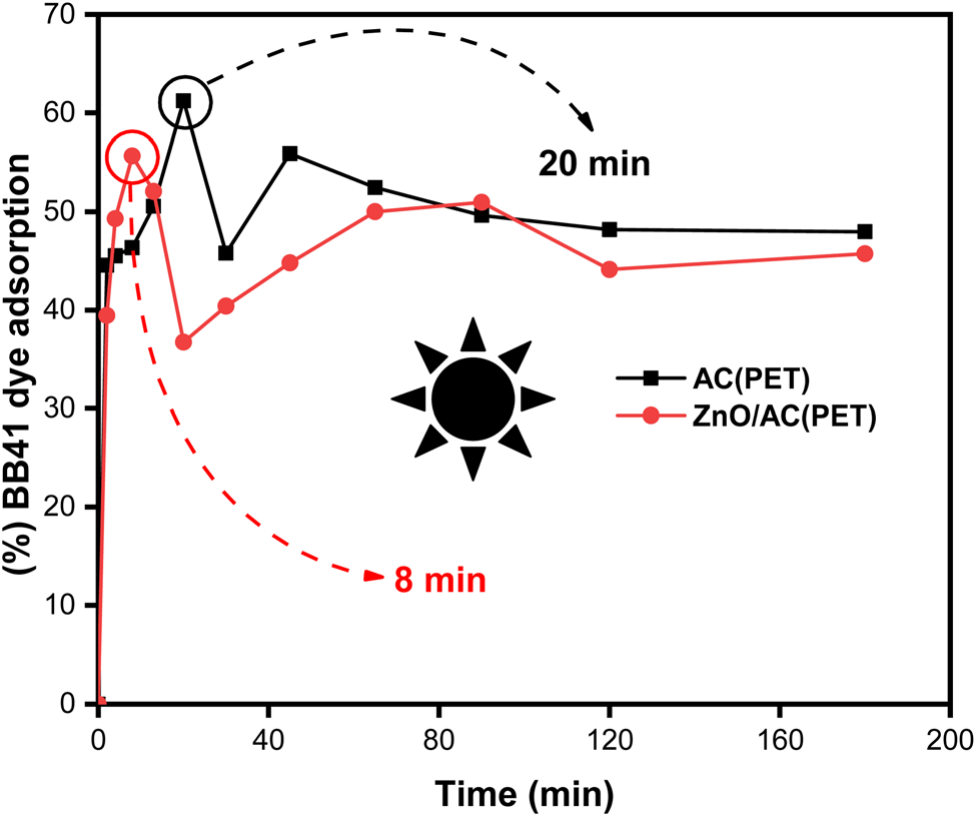
Effect of contact time on BB41 adsorption.

Fig. 5 compares the adsorption of BB41 dye by ZnO/AC(PET) and AC(PET). The results show that the maximum adsorption efficiency is reached at 20 min for AC(PET) (61.22%) and at 8 min for ZnO/AC(PET) (55.66%). After these times, slight desorption is observed for both materials, indicating a reversible interaction between the dye and the adsorbent. Although the overall adsorption efficiency of the ZnO/AC(PET) composite is slightly lower than that of AC(PET) alone, it reaches its maximum adsorption much faster due to the presence of ZnO nanoparticles partially occupying the pores of the activated carbon, which reduces the specific surface area available for adsorption.

### 3.3. Effect of ZnO/AC(PET) photocatalyst dose

**Fig. 6:**
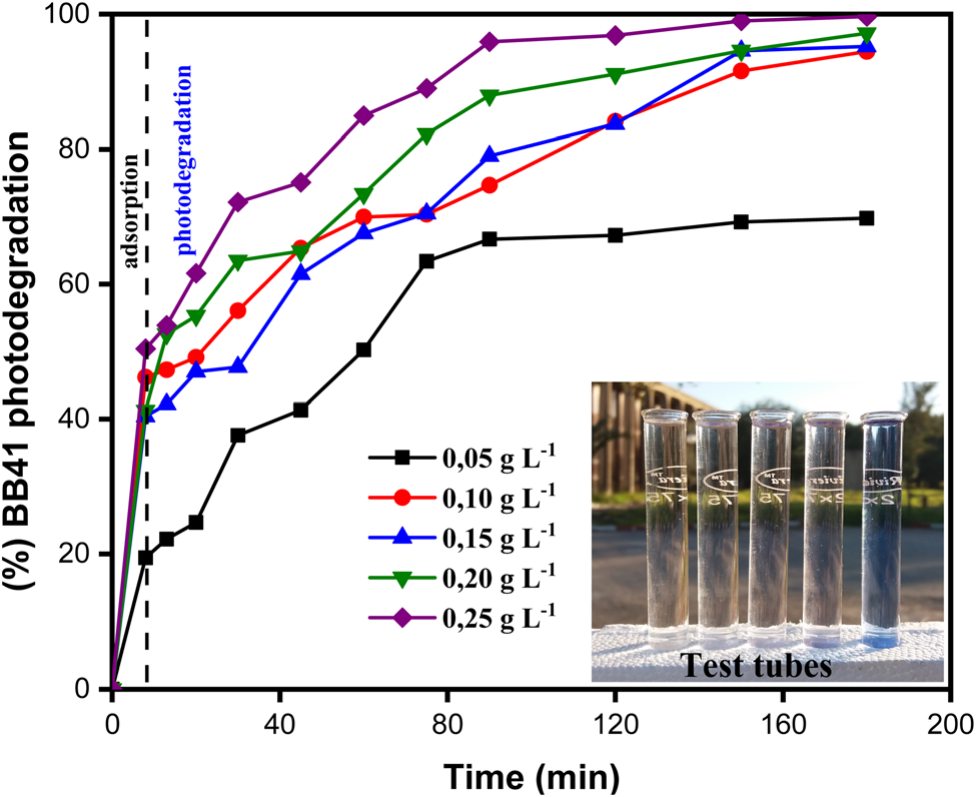
Influence of ZnO/AC(PET) dose on BB41 dye photodegradation efficiency.

**Fig. 7:**
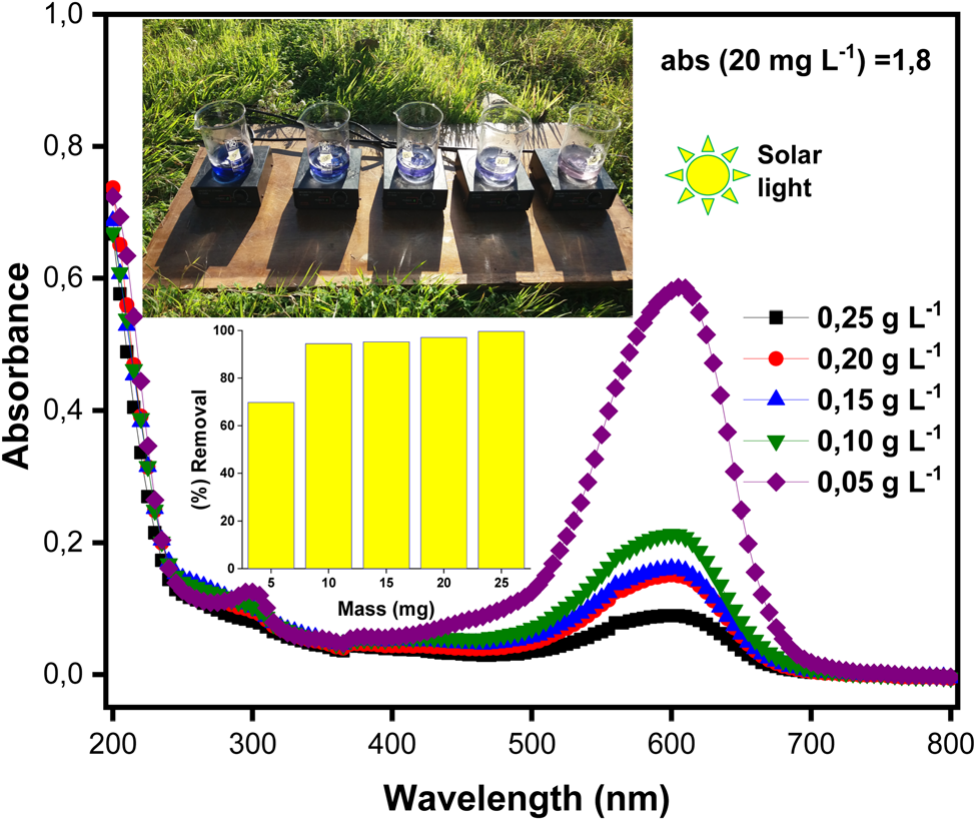
UV-visible absorption spectra of BB41 dye photodegradation at different doses of ZnO/AC(PET).

The analysis of the ZnO/AC(PET) heterosystem shows two distinct stages in the removal of BB41 dye: initial adsorption in the dark, followed by photodegradation under solar irradiation (Fig. 6). The porous structure of activated carbon promotes rapid adsorption within the first eight minutes, allowing partial dye retention. The efficiency of BB41 removal depends on the mass of the ZnO/AC(PET) catalyst, reaching 50.39% for 25 mg of the hybrid material. Upon exposure to sunlight, photocatalysis dominates, as ZnO generates electron–hole pairs (e^−^/h^+^) that produce hydroxyl radicals (OH˙) responsible for dye degradation.^55^ Increasing the mass of ZnO/AC(PET) generally enhances the generation of oxidizing radicals, thus improving BB41 removal. The presence of activated carbon may further facilitate the distribution of ZnO and potentially improve charge separation, as suggested in similar carbon-supported ZnO systems in the literature. A slight decrease in yield is observed at intermediate doses (15–25 mg) compared to 10 mg (94.44%), which could be attributed to minor experimental variations or partial aggregation of ZnO/AC(PET) particles that slightly reduce the effective surface area. However, at the highest dose tested (25 mg), the total available surface area and active sites are sufficient to achieve almost complete degradation of BB41 after 80 minutes, demonstrating that the system maintains high efficiency across all tested doses. The final transparent solutions indicate that the process extends beyond simple adsorption and results in near-complete mineralization of the dye. Dark adsorption tests showed that AC(PET)-based sorption accounted for approximately 20–50% of BB41 removal before irradiation, while the subsequent photocatalysis step degraded the remaining fraction. This confirms that the ZnO/AC(PET) system operates through a synergistic mechanism: activated carbon facilitates the rapid pre-concentration of dye molecules, and ZnO nanoparticles drive their photodegradation under solar light. The UV-vis spectra (Fig. 7) further demonstrate that absorbance decreases significantly as the mass of ZnO/AC(PET) increases, with 10 mg identified as the optimal dose for investigating the effect of the initial BB41 concentration, yielding more than 94% removal and a residual concentration of 1.11 mg L^−1^.

### 3.4. Effect of the initial concentration of BB41

**Fig. 8:**
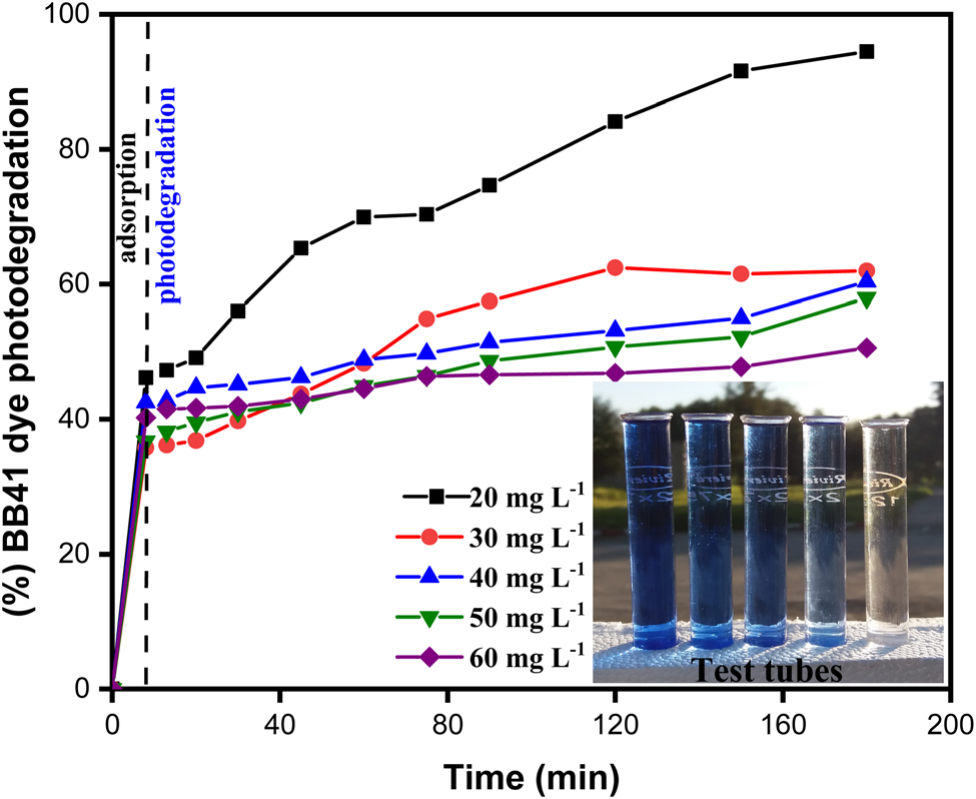
Effect of the initial BB41 dye concentration on photodegradation by ZnO/AC (PET).

**Fig. 9:**
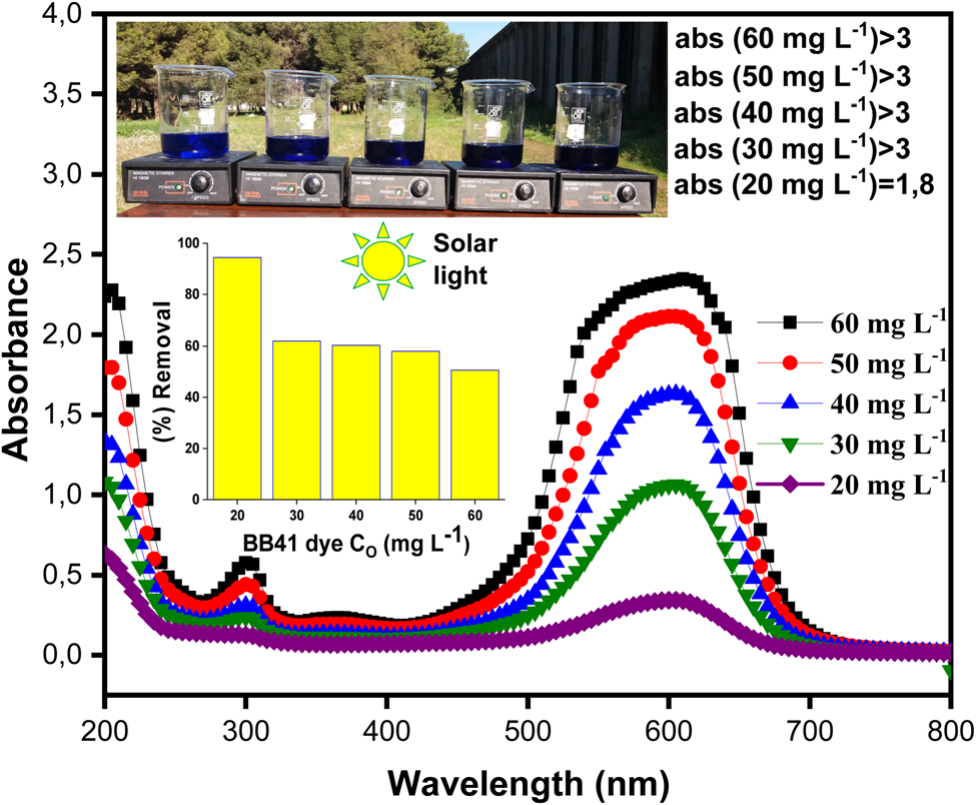
UV-visible absorption spectra of the (20, 30, 40, 50, and 60 mg L^−1^) BB41 dye solutions after photodegradation.

Fig. 8 ^’^s kinetic data demonstrate that the dye's initial concentration significantly affects the photocatalytic process's efficiency. Because more active sites are available and there is less rivalry between dye molecules for adsorption and oxidation, photodegradation is more effective at low concentrations (20–30 mg L^−1^), reaching high rates; a very high photodegradation efficiency of 94.44% was obtained for 20 mg L^−1^. The effectiveness of photodegradation diminishes at higher concentrations (40–60 mg L^−1^) despite an increase in the amount of adsorbed dye. This is likely because of both catalytic site saturation and the self-screening effect of the dye that restricts the doped ZnO/AC(PET) 's ability to absorb solar energy.^56,57^ These findings were supported by the spectrophotometric measurements of the solutions (Fig. 9) following the photocatalytic reaction. The absorbance spectra revealed a notable reduction in the BB41-specific peaks, indicating progressive dye degradation. The trends observed in the kinetic results are validated by comparing the final spectra for each initial concentration, which also demonstrates that photodegradation functions better at lower dye concentrations. For initial concentrations of 20, 30, 40, 50, and 60 mg L^−1^, the adsorption capacities at *t* = 8 min are 92.33, 108.44, 171.11, 205.77, and 248.88 mg g^−1^, respectively. This suggests that the higher the dye concentration, the more the material is adsorbed because a stronger concentration gradient favors interaction with functionalized ZnO/AC(PET) active sites.

### 3.5. Langmuir and Freundlich models

The Langmuir and Freundlich models were used to evaluate the adsorbent's affinity and the type of surface contacts in order to characterize the adsorption mechanism of BB41 dye by the produced ZnO/AC(PET). The Freundlich model considers adsorption on a heterogeneous surface with sites of varying energies, while the Langmuir model assumes adsorption on a homogenous monolayer with a finite number of active sites. We can determine which model best describes the phenomenon being studied by fitting the experimental data to both models. Below are the linearized equations for these models.^58,59^

Linear Langmuir model3
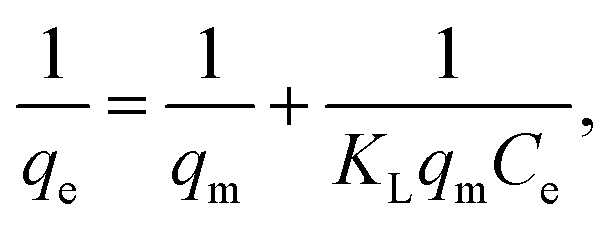
where *C*_e_ (mg L^−1^) is the amount of adsorbate remaining in the solution at equilibrium, *q*_e_ (mg g^−1^) is the amount of adsorbate (mg) adsorbed per unit of adsorbent (g), *q*_m_ (mg g^−1^) is the maximum adsorption capacity, and *K*_L_ (L mg^−1^) is the Langmuir isotherm constant. The graph of the Langmuir isotherm is represented by the plot of 1/*q*_e_ as a function of 1/*C*_e_, where the slope is 1/(*q*_m_*K*_L_) and 1/(*q*_m_) is the *y*-intercept.

**Fig. 10:**
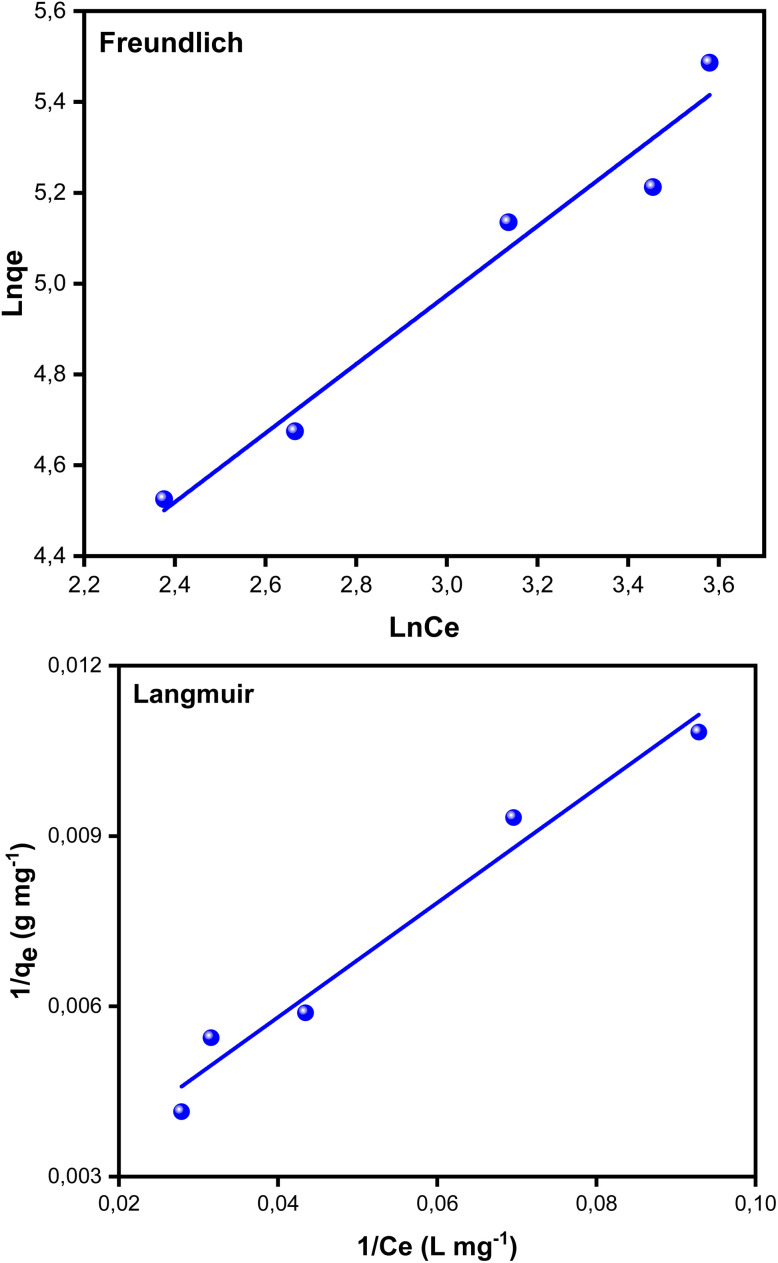
Linearized Langmuir and Freundlich isotherms for BB41 dye adsorption.

Linear Freundlich model4
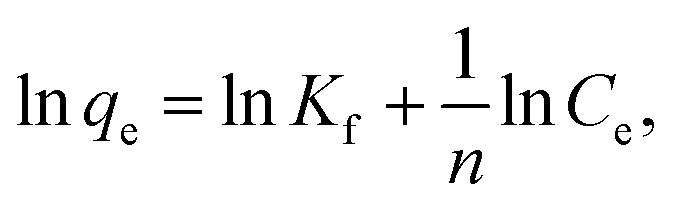
where *q*_e_ denotes the amount of adsorbate (mg g^−1^) adsorbed on the adsorbent at equilibrium, *C*_e_ represents the concentration of the solution at equilibrium (mg L^−1^), *K*_f_ is the adsorption capacity (mg L^−1^) of the adsorbent, and *n* denotes the adsorption intensity. The Freundlich adsorption isotherm graph is plotted between ln *q*_e_ and ln *C*_e_ to help calculate the adsorption capacity and adsorption intensity using the intercept (ln *K*_f_) and slope (1/*n*), respectively. We examine how well the Langmuir and Freundlich models suit the BB41 dye's adsorption by the improved ZnO/AC(PET) material based on the graphs shown in Figure10 and Table 1. A slightly higher correlation coefficient (*R*^2^) for the Langmuir model (*R*^2^ = 0.9628) than for the Freundlich model (*R*^2^ = 0.9524) indicates that the Langmuir model fits the data better. Furthermore, the Langmuir model yielded a maximum adsorption capacity *q*_m_ of 561.80 mg g^−1^, suggesting that the heterosystem ZnO/AC(PET) material has a strong affinity for BB41 dye. Favorable adsorption is confirmed using the Freundlich model, where parameter *n* is greater than 1 (*n* = 1.3162). The Langmuir model's superior correlation, however, indicates that adsorption primarily occurs as a homogenous monolayer over energetically equivalent active sites. Therefore, the adsorption of the BB41 dye onto the ZnO/AC hybrid material is better described by applying the Langmuir model.

**Table 1:** Parameters of the Langmuir and Freundlich isotherms for the adsorption of the BB41 dye by ZnO/AC(PET)

Model	Parameters	Values
Langmuir	*q*_m_(mg g^−1^)	561.80
	*K*_L_ (L mg^−1^)	0.0177
	*R* ^2^	0.9628
Freundlich	*K*_F_	14.811
	*n*	1.3162
	*R* ^2^	0.9524

### 3.6. Kinetic models

To ascertain which of the two conventional kinetic models, the first-order and second-order models, best captures the process, we assessed the kinetics of BB41 dye photodegradation by applying the ZnO/AC(PET) system in this work. Plotting ln(*C*_o_/*C*) for the first-order model and 1/*C* for the second-order model as a function of time allowed us to analyze linearity and correlation coefficients. After selecting the best model, we further interpreted the data using the Langmuir–Hinshelwood (L–H) model, which verified that the reaction was constrained by the BB41 dye's photocatalysis on the composite ZnO/AC(PET). Below are the equations for the kinetic models used in this study, including the first-order model, second-order model and Langmuir–Hinshelwood model.^60–63^

Linear first order model5
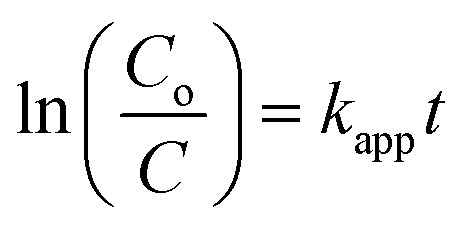


Linear second order model6
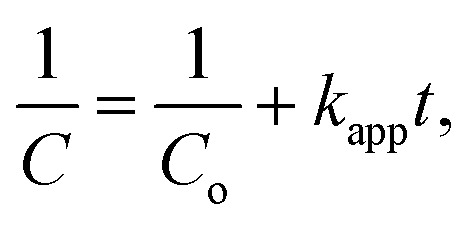
where *C*_o_ is the initial dye concentration (mg L^−1^), *C* is the concentration at time *t* (mg L^−1^), *k*_app_ is the apparent kinetic constant (min^−1^) and *t* is the irradiation time (min).

Linear Langmuir–Hinshelwood model7
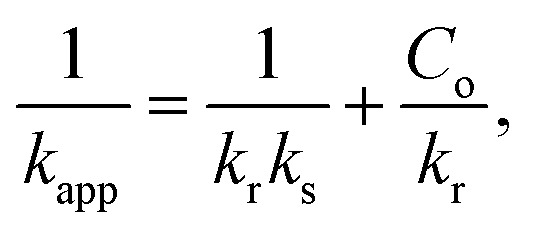
where *k*_r_ and *k*_s_ are the intrinsic reaction constant (min^−1^) and the adsorption constant (L mg^−1^), respectively.

**Fig. 11:**
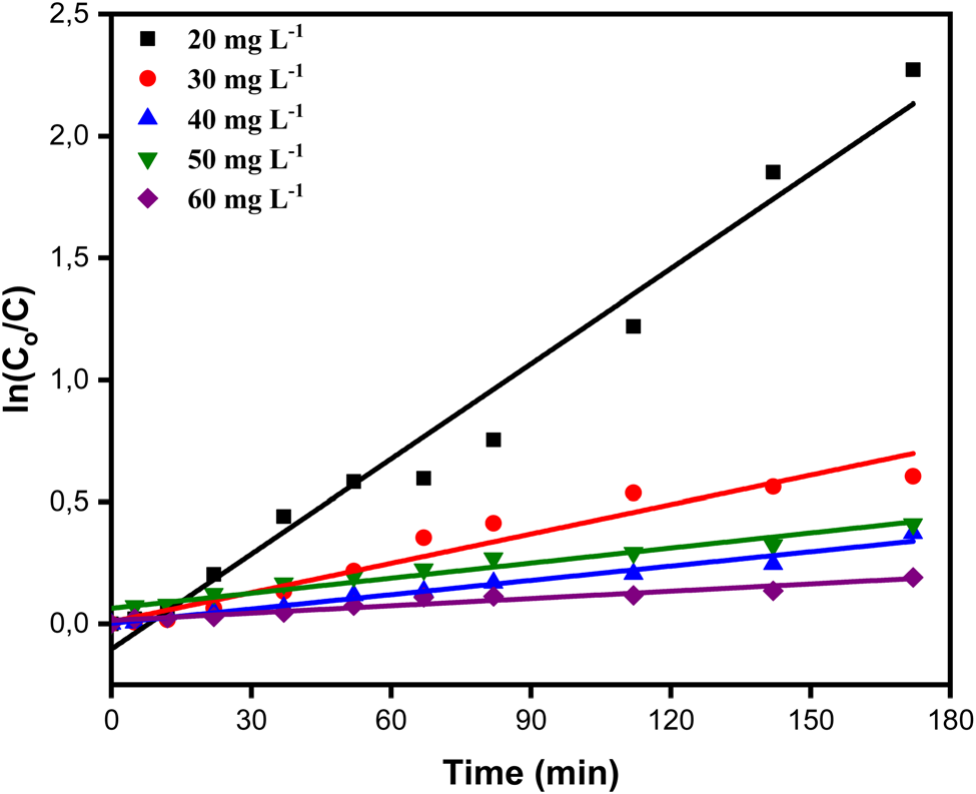
First-order kinetics of BB41 dye degradation by catalytically active ZnO/AC(PET) at different concentrations.

**Fig. 12:**
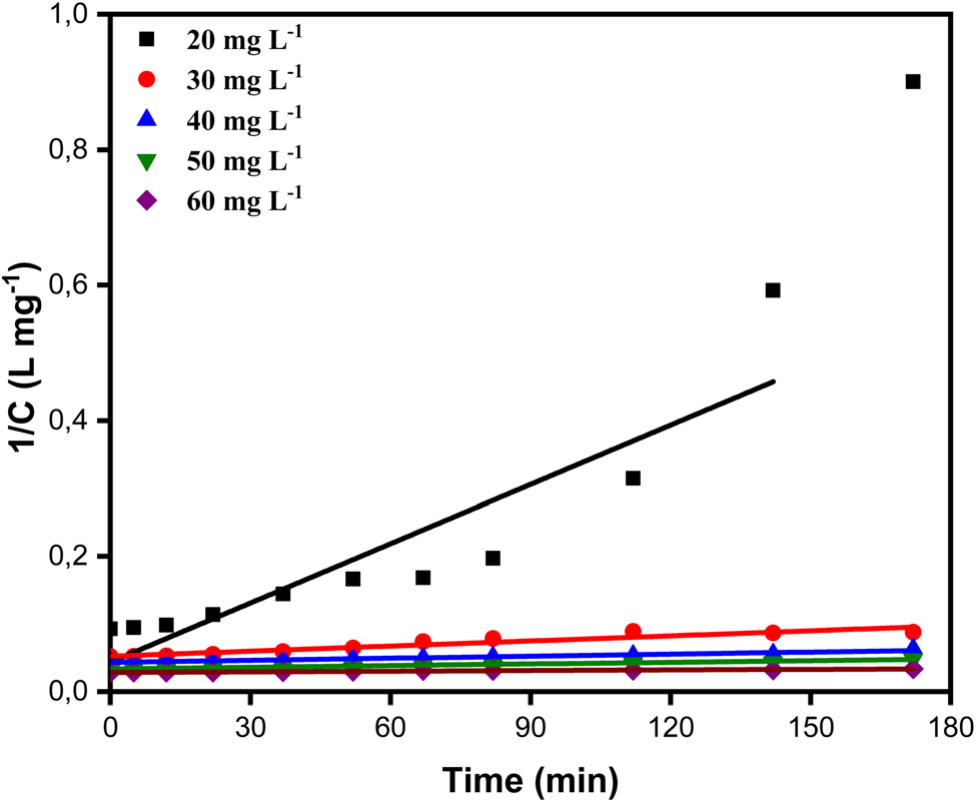
Second-order kinetics of BB41 dye degradation by catalytically active ZnO/AC(PET) at different concentrations.

**Fig. 13:**
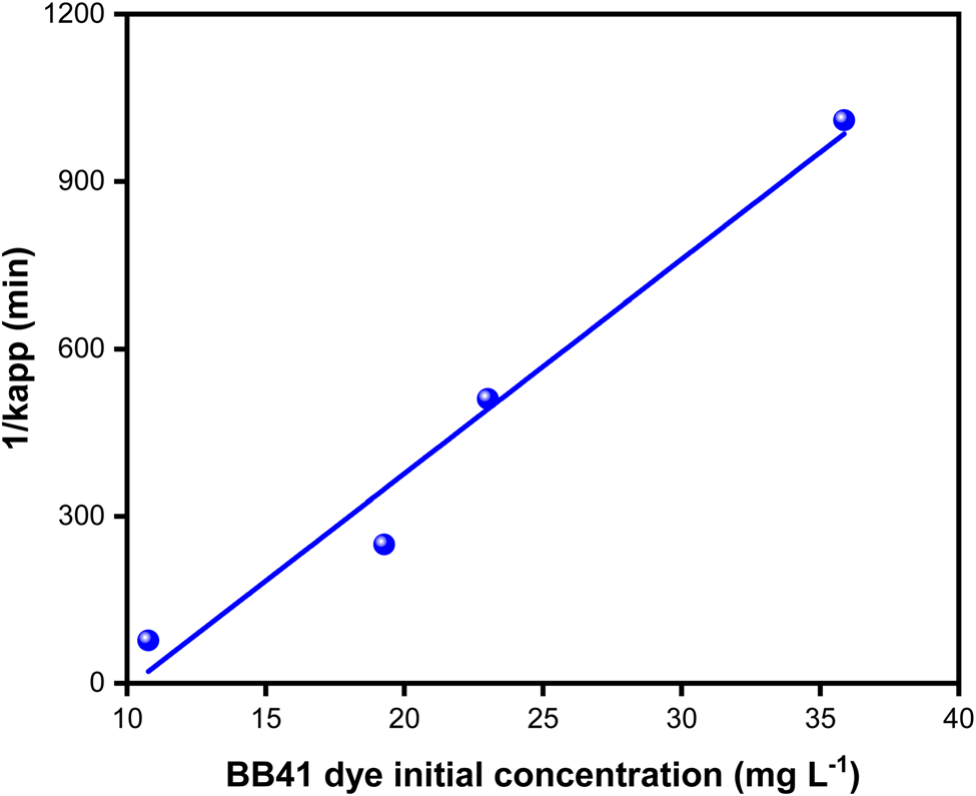
Langmuir–Hinshelwood plot of 1/*k*_app_ against the initial concentration of the BB41 dye.

The results in Fig. 11 and 12 and Table 2 show that the first-order model provides a better fit, with high correlation coefficients. For example, *R*^2^ = 0.9729 for *C*_o_ = 10.767 mg L^−1^*versus R*^2^ = 0.7962 for the second-order and *R*^2^ = 0.9739 for *C*_o_ = 23.011 mg L^−1^*versus R*^2^ = 0.9603. This suggests that the rate of photodegradation is related to the solution concentration and follows an exponential law. Additionally, as *C*_o_ increases, the apparent kinetic constant *k*_app_ gradually decreases, going from 0.01300 min^−1^ at 10.767 mg L^−1^ to just 0.00099 min^−1^ at 35.867 mg L^−1^. This trend suggests the saturation of the material's active sites or an auto-inhibition effect, which is typical of the Langmuir–Hinshelwood model.^64^ Plotting 1/*k*_app_ as a function of *C*_o_ is required to verify linearity and extract adsorption and reaction parameters, which is important to validate this hypothesis. With an adsorption constant *K*_s_ = 0.0260 L mg^−1^ and an intrinsic reaction constant *K*_r_ = 0.09787 m^−1^, the Langmuir–Hinshelwood model findings shown in Fig. 13 verify that adsorption on efficient ZnO/AC(PET) limits the photodegradation of BB41 dye. A high correlation coefficient *R*^2^ = 0.95823 indicates that the data fit this model well, supporting the idea that the process is governed by the interaction between the dye and the active surface. As previously indicated by the analysis of the first-order model, these results are in line with the observed decrease in *k*_app_ as *C*_o_ increases, reflecting the gradual saturation of the active sites and adsorption-influenced kinetics.

**Table 2:** Parameters of the pseudo first- and second-order kinetics of photodegradation at various initial concentrations of the BB41 dye

Model	Parameters and values			
	*C*_0_ (mg L^−1^)	*C* (mg L^−1^) after adsorption	*k*_app_(min^−1^)	*R* ^2^
First order	20	10.767	0.01300	0.9729
	30	19.278	0.00401	0.9497
	40	23.011	0.00196	0.9739
	50	31.644	0.00206	0.9464
	60	35.867	0.00099	0.9493
Second order	20	10.767	0.00292	0.7962
	30	19.278	0.00025	0.8998
	40	23.011	0.00010	0.9603
	50	31.644	0.00008	0.9574
	60	35.867	0.00003	0.9427

### 3.7. ROS trapping studies

**Fig. 14:**
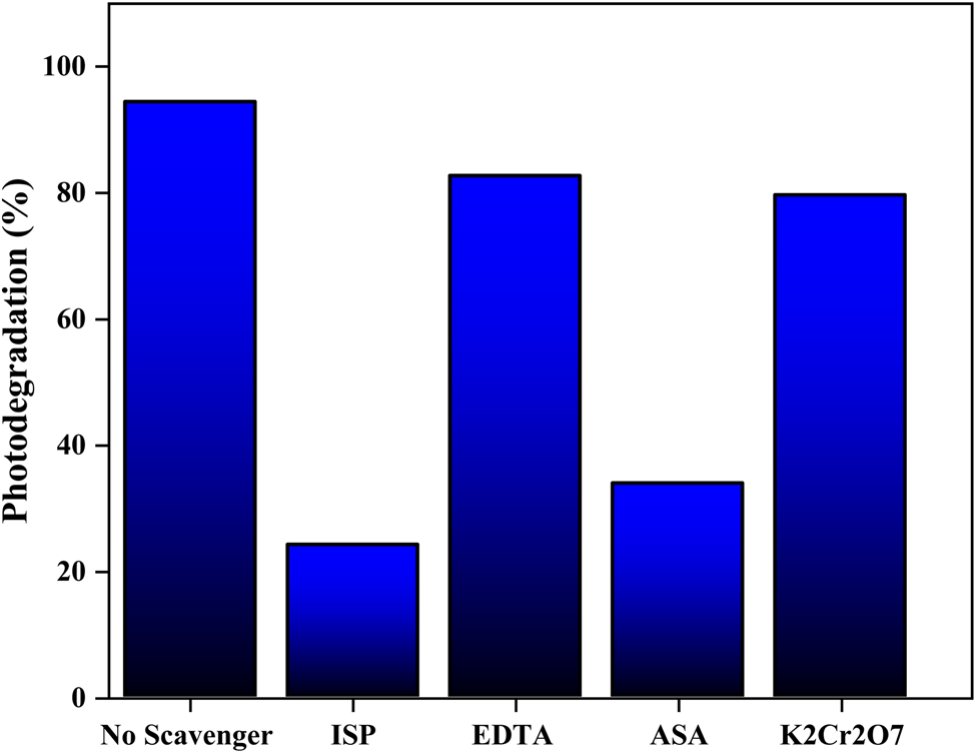
Effects of scavengers on the photodegradation of BB41 over ZnO/AC.

To gain insight into the photocatalytic mechanism of BB41 degradation over ZnO/AC, a series of scavenger experiments was performed under optimized conditions (BB41: 20 mg L^−1^, ZnO/AC: 0.1 g L^−1^), and the results are shown in Fig. 14. In the absence of any scavenger, the system achieved a degradation efficiency of 94.48%, confirming the strong photocatalytic activity of the composite. The addition of isopropanol (IPA), a selective scavenger for hydroxyl radicals (˙OH), caused a sharp decrease in degradation efficiency to 24.1%, highlighting the critical role of ˙OH as the main oxidative species responsible for dye breakdown. Similarly, introducing ascorbic acid (ASA), which targets superoxide radicals (O_2_˙^−^), also significantly reduced the degradation to 33.9%, demonstrating that O_2_˙^−^ radicals also play a major role in the degradation pathway. In contrast, when potassium dichromate (K_2_Cr_2_O_7_) was used to capture photogenerated electrons (e^−^), the degradation dropped moderately to 79.6%, suggesting that electrons contribute to ROS generation but are not the primary actors in direct pollutant degradation. The addition of EDTA, a known hole (h^+^) scavenger, led to a degradation efficiency of 82.7%, indicating that holes are involved, but to a lesser extent compared to ˙OH and O_2_˙^−^ radicals. These results imply that the photocatalytic degradation of BB41 is mainly driven by hydroxyl and superoxide radicals, which are produced through successive redox reactions involving photogenerated charge carriers. Under irradiation, ZnO absorbs photons, promoting electrons to the conduction band and leaving holes in the valence band. The conduction band electrons reduce dissolved O_2_ to generate O_2_˙^−^, while the holes oxidize water or surface hydroxyl groups to form ˙OH. These ROS collectively initiate oxidative attacks on BB41 dye molecules, leading to mineralization. Under light irradiation, the ZnO/AC photocatalyst absorbs photons with energy equal to or greater than its band gap. This excitation promotes electrons (e^−^) from the valence band to the conduction band, simultaneously generating positively charged holes (h^+^) in the valence band.

### 3.8. Photocatalyst reusability and stability

**Fig. 15:**
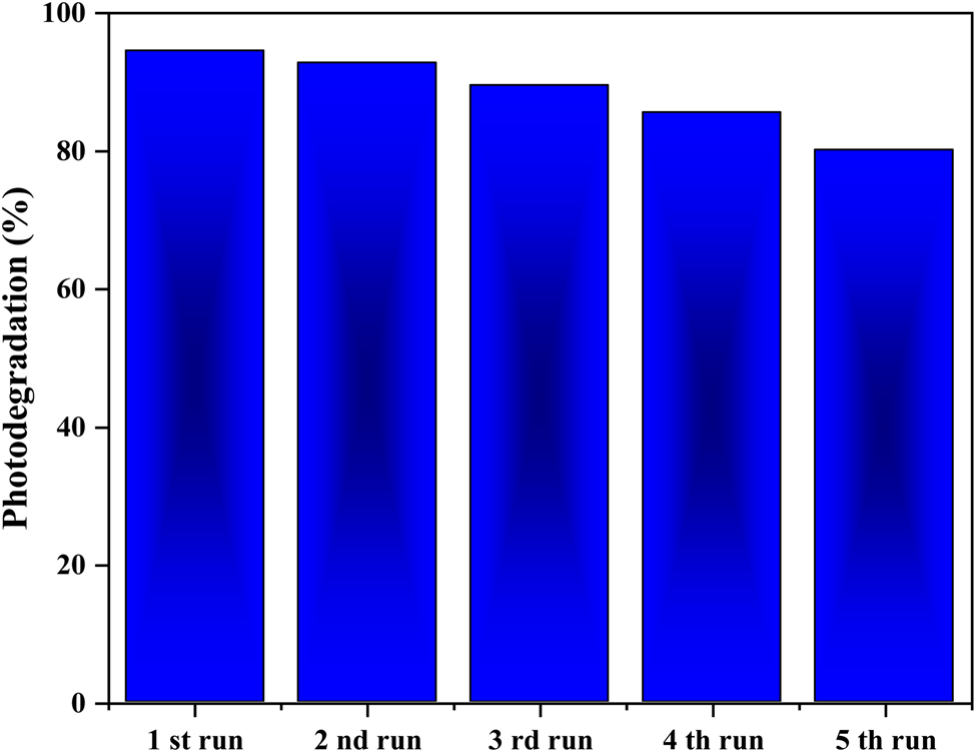
Reusability of the ZnO/AC(PET) photocatalyst for BB41 degradation under solar irradiation.

Catalyst recyclability is a critical factor in evaluating the practical applicability of heterogeneous photocatalysts for wastewater treatment. The ZnO/AC(PET) photocatalyst was recovered after each cycle and reused for successive BB41 degradation experiments under solar irradiation (Fig. 15). A gradual decrease in photocatalytic performance was observed, with the degradation efficiency decreasing to approximately 80% after five consecutive cycles. The good reusability of ZnO/AC(PET) can be attributed to its easy recovery from the aqueous medium, which occurs through the rapid sedimentation of the photocatalyst within less than 15 minutes after each cycle. The slight loss of activity may be associated with partial surface deactivation caused by the accumulation of intermediate species on active sites.^65,66^

### 3.9. Comparison with previously reported ZnO/AC-based materials

Table 3 presents a comparative analysis of previously reported ZnO/AC-based materials for dye degradation. Although high removal efficiencies have been reported in the literature, these performances have often been achieved using significantly higher catalyst dosages or under artificial UV irradiation. In contrast, the ZnO/AC(PET) composite developed in this work achieved 95% removal of BB41 at a relatively low catalyst dosage (100 mg L^−1^) under natural sunlight, demonstrating its potential as a cost-effective and sustainable alternative to wastewater treatment.

**Table 3:** Comparison of ZnO/AC-based materials for photocatalytic dye degradation

Materials	Dye, *C*_o_ (mg L^−1^)	Materials dosage (mg L^−1^)	Irradiation time (min)-light source	Removal (%)	References
ZnO/AC	MB: 80	250	45 min-UV	92%	67
ZnO/AC	MB: 100	3000	180 min-visible	99%	68
ZnO/AC	MG: 22	500	120 min-sunlight	100%	69
ZnO/AC	CR: 21	500	80 min-sunlight	100%	69
ZnO/AC	RhB: 5	1400	180 min-Sunlight	86%	70
ZnO/AC (PET)	BB41: 20	100	180 min-Sunlight	95%	This work

### 3.10. Proposed photodegradation mechanism

The ZnO/AC composite shows enhanced photocatalytic activity due to the synergistic interaction between ZnO and activated carbon (AC) under solar light. Upon irradiation, ZnO absorbs photons, exciting electrons from the valence band (VB) to the conduction band (CB) and creating electron–hole pairs:ZnO/AC + *hν* → ZnO/AC (CB e^−^) + ZnO/AC (VB h^+^)

Electrons (e^−^) in the CB react with molecular oxygen (O_2_) to form superoxide radicals (O_2_˙^−^).ZnO/AC (CB e^−^) + O_2_ → O_2_˙^−^

Holes (h^+^) in the VB oxidize water or hydroxide ions to produce hydroxyl radicals (OH˙):ZnO/AC (VB h^+^) + H_2_O → OH˙ + H^+^ZnO/AC (VB h^+^) + OH^−^ → OH˙

These reactive species (O_2_˙^−^, OH˙, h^+^) attack BB41 molecules, degrading them into CO_2_, H_2_O, and minor by-products:BB41 + OH˙ + O_2_˙^−^ + h + → CO_2_ + H_2_O + by-products.

**Fig. 16:**
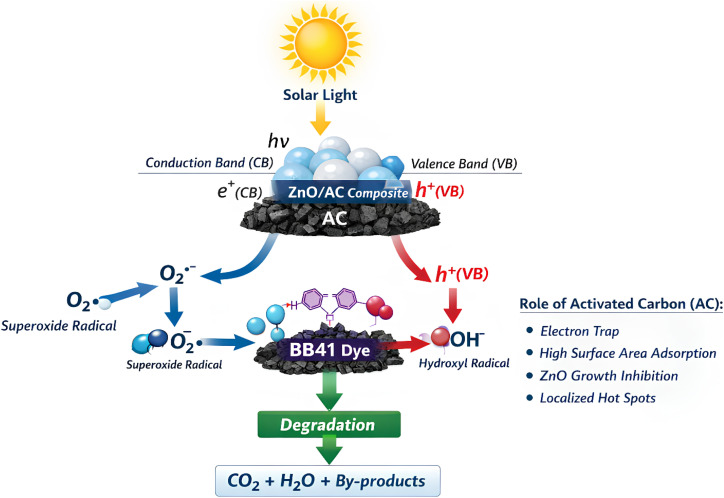
Schematic of the BB41 photodegradation mechanism over ZnO/AC under solar irradiation.

Fig. 16 illustrates the proposed photocatalytic mechanism of BB41 degradation over the ZnO/AC composite under solar light, highlighting the generation of reactive species and the role of activated carbon in enhancing the process.

### 3.11. Photocatalytic performance toward various cationic dyes

**Fig. 17:**
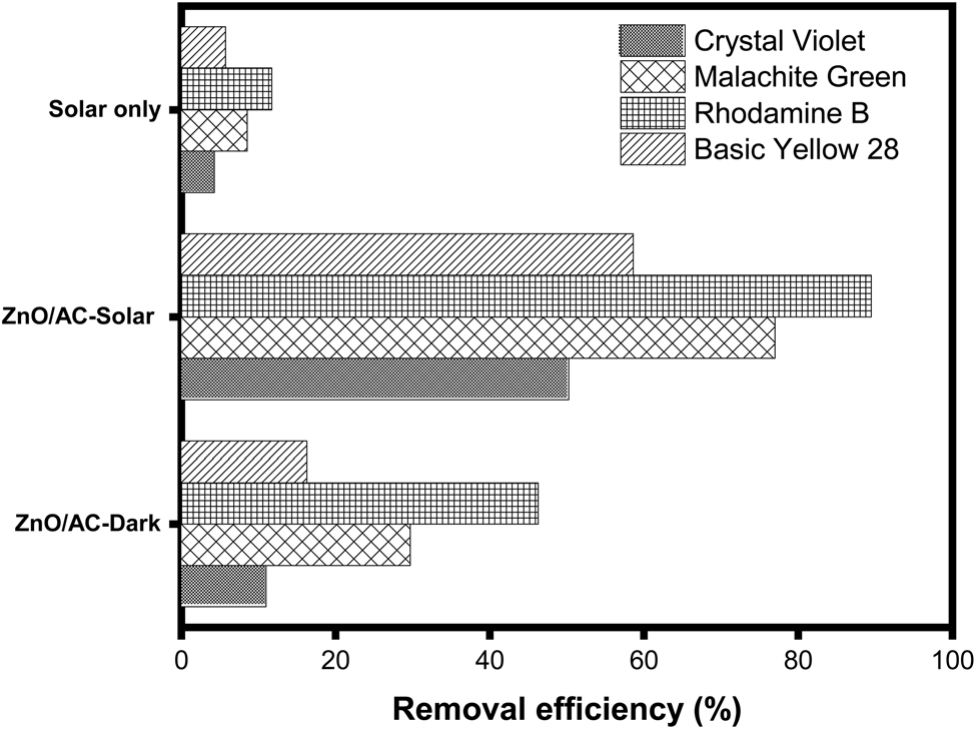
Adsorption, photolysis and photocatalytic degradation of cationic dyes.

To further validate the broad applicability of the ZnO/AC(PET) composite, additional photocatalytic experiments were conducted using representative cationic dyes, namely crystal violet (CV), malachite green (MG), rhodamine B (RhB), and basic yellow 28 (BY28). All experiments were performed under the same optimized conditions previously established for BB41 degradation, including an initial dye concentration of 20 mg L^−1^, catalyst dosage of 10 mg in 100 mL solution (0.1 g L^−1^), natural pH without adjustment, and a total contact time of 180 min under adsorption and solar irradiation. Fig. 17 illustrates the comparative degradation efficiencies of various cationic dyes under different conditions, highlighting the dominant role of photocatalysis over adsorption and photolysis. Control experiments carried out under solar irradiation without a catalyst revealed limited degradation efficiencies (4.28–11.73%), indicating that direct photolysis plays a minor role. In contrast, the ZnO/AC(PET) composite exhibited significantly enhanced removal efficiencies, reaching 50.23%, 76.98%, 89.47%, and 58.61% for CV, MG, RhB, and BY28, respectively. Additionally, adsorption for 180 min under dark conditions (10.98–46.28%) confirms the contribution of the activated carbon component. The superior performance under solar irradiation highlights the synergistic effect between adsorption and photocatalysis. The variation in degradation efficiency among dyes can be attributed to differences in molecular structures, light absorption properties, and interactions with the catalyst surface. Overall, these results clearly demonstrate the effectiveness and versatility of ZnO/AC(PET) for the removal of various cationic organic pollutants under realistic conditions.

## 4. Conclusion

In this study, a straightforward impregnation method was used to create a supported ZnO nanoparticle catalyst on activated carbon. FTIR spectrometric analysis revealed the presence of Zn–O functional groups on the synthesized material in the region of 500–400 cm^−1^. ZnO-impregnated activated carbon (PET) has the same characteristic peaks as crystalline ZnO in its X-ray diffractogram but at a lesser intensity. This functionalized ZnO/AC(PET) was tested to eliminate blue basic 41 (BB41), a synthetic dye present in the effluents of the textile and tanning industries. The dye in the solution was treated using two methods: a physicochemical process and an advanced oxidation process involving adsorption and solar photocatalysis. The maximum adsorption time recorded by ZnO/AC(PET) was 8 minutes, with significant adsorption capacities; the residual concentration after adsorption was eliminated by photocatalysis using the ZnO nanoparticles present on the activated carbon. This process takes only 180 minutes to reach its maximum. The hybrid treatment system confirms the ability and performance of the developed material to effectively eliminate the target molecule (BB41 dye). The kinetic results validate its potential as a high-performance photocatalyst. In order to get around ZnO's large band gap, future studies should concentrate on doping or surface modification techniques to increase the dispersion of ZnO on AC(PET). They should also investigate visible light-driven photocatalysis. To evaluate the practical applicability of the composite, its long-term stability and regeneration ability were investigated through several regeneration cycles. The results demonstrated that the material maintained satisfactory photocatalytic performance after successive use, indicating good stability and reusability. Furthermore, more realistic insights into process efficiency could be obtained by extending the investigation from model dye solutions to real wastewater samples. Finally, techno-economic and environmental assessments are still necessary to determine the feasibility of scaling up this hybrid treatment strategy.

## Author contributions

Dahdouh Nadjib: conceptualization, methodology, investigation, data curation, writing – original draft, writing – review & editing, supervision, project administration. Hafsa Haroun: investigation, data curation, writing – original draft. Djemmali Badreddine: formal analysis, validation. Ouarghi Amina: investigation, resources. Kezrane Imane: investigation. Chaabane Toufik: visualization, data curation. Sivasankar Venkataraman: writing – review & editing, validation. Kebir Mohammed: supervision.

## Conflicts of interest

The authors declare that they have no known competing financial interests or personal relationships that could have appeared to influence the work reported in this paper.

## Data Availability

The data supporting the findings of this study are available from the corresponding author upon reasonable request.
